# Transition-Metal-Doped Nickel–Cobalt Layered Double Hydroxide Catalysts for an Efficient Oxygen Evolution Reaction

**DOI:** 10.3390/ma18040877

**Published:** 2025-02-17

**Authors:** Zhihan Li, Wenjing Yi, Qingqing Pang, Meng Zhang, Zhongyi Liu

**Affiliations:** 1 College of Chemistry, Zhengzhou University, Zhengzhou 450001, China; 2 School of Chemical Engineering, Zhengzhou University, Zhengzhou 450001, China; 3 State Key Laboratory of Coking Coal Resources Green Exploitation, Zhengzhou University, Zhengzhou 450001, China

**Keywords:** layered double hydroxide, transition metal, doping, hydrogen, oxygen evolution reaction

## Abstract

Hydrogen plays a vital role in the global shift toward cleaner energy solutions, with water electrolysis standing out as one of the most promising techniques for generating hydrogen. Despite its potential, the oxygen evolution reaction (OER) involved in this process faces significant challenges, including high overpotentials and slow reaction rates, which underscore the need for advanced electrocatalytic materials to enhance efficiency. Noble metal catalysts are effective but expensive, so transition-metal-based electrocatalysts like nickel–cobalt layered double hydroxides (NiCo LDHs) have become promising alternatives. In this research, a series of NiCo LDH catalysts doped with Fe, Mn, Cu, and Zn were effectively produced using a one-step hydrothermal technique. Among the catalysts, the Fe-doped NiCo LDH exhibited OER activity, achieving a lower overpotential (289 mV) at a current density of 50 mA/cm^2^, which was far better than the 450 mV of the undoped NiCo LDH. The Mn-, Cu-, and Zn-NiCo LDHs also exhibited lower overpotentials of 414 mV, 403 mV, and 357 mV, respectively, at this current density. The Fe-doped NiCo LDH had a 3D layered nanoflower structure, increasing the surface area for reactant adsorption. The electrochemically active surface area (ECSA), as indicated by the double-layer capacitance (C_dl_), was larger in the doped samples. The C_dl_ value of the Fe-doped NiCo LDH was 3.72 mF/cm^2^, significantly surpassing the 0.82 mF/cm^2^ of the undoped NiCo LDH. These changes improved charge transfer and optimized reaction kinetics, enhancing the overall OER performance. This study offers significant contributions to the development of efficient electrocatalysts for the OER, advancing the understanding of key design principles for enhanced catalytic performance.

## 1. Introduction

Hydrogen has been increasingly recognized as a vital element in the transition to sustainable energy systems [1]. One of the primary advantages of hydrogen is its capacity to be produced from a variety of sources, including renewable energy through water electrolysis [2,3]. This method is particularly appealing since it generates hydrogen without carbon emissions, aligning with the goal of global climate change [4].

The oxygen evolution reaction (OER) holds a pivotal position among the various electrochemical processes. It is a crucial half-reaction occurring specifically at the anode during the intricate process of water electrolysis. This reaction is characterized by a significant energy barrier and sluggish kinetics, making it the rate-limiting step in the water splitting process [5,6]. The efficiency of the OER is significantly influenced by the choice of electrocatalysts, which speed the reaction by lowering the activation energy required for the process [7]. Noble metal catalysts play a main role in electrocatalysis due to their exceptional catalytic properties [8,9]. Their significant reactivity, stability, and capacity to accelerate electron transfer render them essential for advancing effective energy conversion and storage technologies [10]. However, the high cost and shortage of noble metals pose significant challenges for large-scale commercialization.

Transition metal electrocatalysts can play an alternative role in various reactions, particularly in energy conversion processes such as the OER [11,12]. These materials are favored for their unique properties, including high catalytic activity, stability, and the ability to be synthesized from abundant and inexpensive elements [13]. Among these materials, transition-metal-layered double hydroxides (TM-LDHs) stand out as particularly efficient electrocatalysts for the oxygen evolution reaction (OER), especially the (Ni, Co, Fe)-LDHs. This efficiency largely stems from their distinctive layered structures and substantial specific surface area, which provide an increased number of accessible active sites, enhance anion exchange abilities, and facilitate improved charge and mass transport characteristics. Such properties render LDHs immensely useful across various applications [14,15,16].

The electrolysis process enables water molecules or hydroxyl ions to be incorporated into the layered structure of layered double hydroxides (LDHs). This incorporation enhances the proton-coupled electron transfer mechanism, which is essential for enhancing the overall catalytic efficiency of LDHs. As a result, the interaction between these molecules and the layered structure not only aids in the structural integrity of the materials but also significantly boosts their efficiency in catalytic applications [17,18]. Additionally, nickel–cobalt layered double hydroxides (NiCo LDHs) have been treated as effective electrocatalysts during the OER, especially in alkaline environments [19,20]. The combination of nickel and cobalt in LDHs leads to effects that enhance their electrocatalytic properties. The combination of both metals creates an enhanced electronic environment that can improve the binding energies of reaction intermediates and optimize the OER process [21,22]. This synergy is particularly beneficial to the formation of active high-valence states during the reaction. NiCo LDHs exhibit numerous advantages; however, there still remains significant potential for enhancing their performance.

Using hydrothermal methods to synthesize NiCo LDHs can achieve better crystallinity and more uniform particle size, which are beneficial for catalytic activity. This particular method offers a remarkable advantage in that it enables meticulous and exacting control over both the composition and morphology of LDHs. When it comes to composition, it allows researchers to fine-tune the elemental ratios within the LDH structure [23]. The morphology design also affects the performance at different current densities. For instance, the hierarchical structure, such as using nickel foam as a base material, can enhance the overall conductivity of the electrode, achieving better charge transfer during the OER process. The self-supported structure not only improves its stability but also allows it to increase the active surfaces that expose more active sites to participate in the reaction [24,25]. Meanwhile, many studies have demonstrated that metal-doped NiCo LDH catalysts can lower overpotentials and accelerate OER kinetics compared to their undoped counterparts [26,27].

Transition metal elements possess abundant electron orbits and exhibit variable oxidation states [28]. This characteristic significantly influences their electronic structure when doped with NiCo LDH. When transition metal atoms are incorporated into the lattice of NiCo LDH, the differences in electronegativity and electron orbital energy levels between these atoms and the Ni and Co atoms lead to a readjustment of the electron cloud distribution [29,30]. This adjustment notably changes the electron cloud density near the active site. The electron cloud is key in chemical reactions as it probabilistically describes electron positions in atoms or molecules, and its distribution around the active site (where reactions occur) is altered upon this adjustment. For example, the nanocages Fe-Co-Ni-LDH need a significantly lower overpotential to reach a specific current density compared to other LDHs, indicating superior catalytic efficiency [31]. When a transition metal is doped, its d-orbital electrons interact with the orbitals of the reactant molecules to establish a specific adsorption mode [32]. If the distribution of electron cloud density is optimized, the reactant molecules can be adsorbed more effectively on the active sites, and the intermediate products generated during the reaction can also be converted more efficiently [33]. This optimization reduces the activation energy of the reaction and significantly enhances the rate of the catalytic reaction.

In this work, we focused on the synthesis of NiCo LDH on nickel foam through a straightforward one-step hydrothermal process. To enhance the catalytic performance, we strategically incorporated other transition metals into the NiCo LDH structure. A series of catalysts with doping elements of Fe, Mn, Cu, and Zn were successfully prepared. Notably, the Fe-NiCo LDH exhibits an impressive overpotential of 289 mV when assessed at 50 mA/cm^2^. This highlights the importance of our doping modification approach in improving the electrocatalytic characteristics of NiCo LDH. The organization of this paper is as follows: Section 1 introduces the background and significance of this study. Section 2 details the experimental methods and materials. Section 3 presents the results and discussion. Finally, Section 4 concludes this study and provides future research directions.

## 2. Experiment

### 2.1. Preparation of the Doped NiCo LDH Catalysts

Before the experiment, the nickel foam (NF) was cleaned with ethanol, 2 mol/L hydrochloric acid, and water via ultrasonic for 15 min and dried in a vacuum at 60 °C. Then, the Ni(NO_3_)_2_ (Aladdin, China), Co(NO_3_)_2_ (Aladdin, China), and a certain amount of FeCl_3_ (Aladdin, China) were dissolved in 30 mL of deionized water. Subsequently, 2 mmol of NH_4_F and 0.5 mmol of urea were added and stirred for 10 min. Finally, the mixture was placed, along with a piece of treated foam nickel (2.5 × 3 cm), into a 50 mL Teflon-lined hydrothermal autoclave and hydrothermally treated at 120 °C for 10 h. Once the reaction finished, the autoclave was cooled to room temperature naturally. The resulting sample underwent the thorough washes with ethanol and deionized water, followed by drying at 60 °C to yield Fe-NiCo LDH. To prepare Mn-NiCo LDH, Cu-NiCo LDH, and Zn-NiCo LDH, FeCl_3_ was replaced by a certain amount of MnCl_2_, CuCl_2_, and ZnCl_2_, respectively.

### 2.2. Structure Characterization

The crystalline structures of the synthesized products were investigated by X-ray diffraction (XRD, Empyrean, PANalytical, Almelo, The Netherlands Cu Kα). The surface chemical states and electronic structures of the prepared products were probed by X-ray photoelectron spectroscopy (XPS, AXIS SUPRA, Shimadzu (Kratos Analytical), Manchester, UK). The morphologies and microstructures of the products were examined by a Scanning Electron Microscope (SEM, ZEISS Sigma 500, Carl Zeiss, Oberkochen, Germany).

### 2.3. Electrochemical Performance Testing

The electrochemical characteristics of the samples were evaluated with an electrochemical workstation (CHI 660e). Electrochemical measurements were performed in a 1.0 M KOH aqueous electrolyte using a conventional three-electrode setup. A carbon electrode functioned as the counter electrode, an Hg/HgO electrode was used as the reference electrode, and the working electrode comprised M-NiCo LDH/NF (M=Fe, Mn, Cu, Zn). Linear sweep voltammetry (LSV) and cyclic voltammetry (CV) were performed. The Nernst equation states that E_RHE_ = E_Hg/HgO_ + (0.098 V + 0.059 V × pH) V; all potentials could be converted into the reversible hydrogen electrodes (RHEs). The overpotential was calculated using the following equation *η* = E_RHE_ − 1.23V.

Linear sweep voltammetry (LSV) measurements were performed at a scan rate of 5 mV s^−1^ to obtain polarization curves. The Tafel slope was calculated by making use of the equation η = b log j + a, where η stands for the overpotential, b refers to the Tafel slope, a is the Tafel intercept, and j is equivalent to the current density (mA cm^−2^). The electrochemical double-layer capacitance (C_dl_) was derived from cyclic voltammetry (CV) curves within a non-Faradaic potential, calculated as C_dl_ = ∆j/∆v, where ∆v refers to the scan rate from 20 to 120 mV s^−1^ and ∆j is defined as half the difference between the anodic and cathodic current densities (∆j = (j_a_ − j_c_)/2).

## 3. Results and Discussion

**Figure 1:**
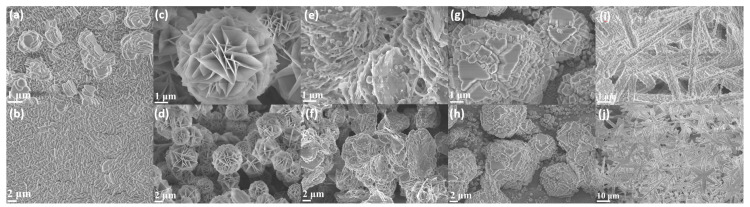
SEM images of (**a**,**b**) NiCo LDH, (**c**,**d**) Fe-NiCo LDH, (**e**,**f**) Mn-NiCo LDH, (**g**,**h**) Cu-NiCo LDH, and (**i**,**j**) Zn-NiCo LDH.

The SEM images depicted in Figure 1a–j reveal the morphological characteristics of the NiCo LDH and its doped variants, including the Fe-NiCo LDH, Mn-NiCo LDH, Cu-NiCo LDH, and Zn-NiCo LDH. These images clearly demonstrate the profound influence of doping on the structural morphology of NiCo LDH. In addition, the Fe-NiCo LDH and Zn-NiCo LDH display distinct morphological features that set them apart from the other samples. The Fe-NiCo LDH features a distinctive 3D layered nanoflower framework (Figure 1c,d). This distinctive structural configuration provides an extensive surface area, which enhances the adsorption capacity for reactant molecules. The hierarchical arrangement of the nanoflowers enables efficient charge transfer during the OER process [34]. The numerous exposed edges and surfaces in this structure serve as active sites, promoting the reaction. The Zn-NiCo LDH presents a 3D thorn-like snowflake structure (Figure 1i,j). The rough and non-uniform surface of the thorns increases the surface area available for the reaction. The unique morphology of the Zn-NiCo LDH contributes to its enhanced electrocatalytic performance in the OER. In contrast, the Mn-NiCo LDH and Cu-NiCo LDH (Figure 1e–h) do not exhibit such well-defined and advantageous morphologies as the Fe-NiCo LDH and Zn-NiCo LDH. This indicates that while doping positively influences the NiCo LDH, a more uniform, open, and three-dimensional morphology offers more significant advantages. These may help present new avenues for further research aimed at enhancing the active sites of catalysts.

**Figure 2:**
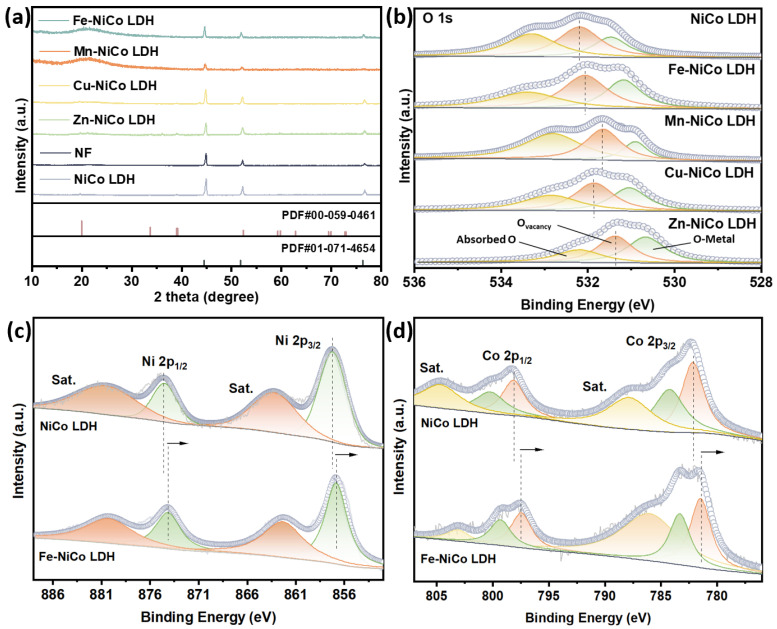
Structural characterization. (**a**) XRD patterns and (**b**) O1s of NiCo LDH, NF, Fe-NiCo LDH, Mn-NiCo LDH, Cu-NiCo LDH, and Zn-NiCo LDH. (**c**) Ni 2p XPS and (**d**) Co 2p XPS of Fe-NiCo LDH.

We characterized the composition and crystalline structure of the synthesized electrocatalysts using X-ray diffraction (XRD). The resulting patterns, as depicted in Figure 2a, closely match the reference data for nickel–cobalt layered double hydroxides (PDF#00-059-0461) and nickel foam (PDF#01-071-4654), confirming the synthesis of the target materials. In comparison to the Cu- and Zn-doped NiCo-LDHs, doping with Fe and Mn leads to more pronounced distortions in the lattice structure, resulting in notable changes to the lattice parameters. The changes in the lattice parameters can be quantitatively analyzed from the shift in the diffraction peaks. Upon doping with Fe, the peak of the Ni-Co LDH shifted to a lower angle. This observation indicates an increase in the interlayer spacing of the Ni-Co LDH structure [35]. This alteration in the lattice structure has the potential to impact the diffusion of reactant ions and the electronic structure of the material. Eventually, it will exert an influence on the catalytic performance. Specifically, lattice distortion modifies the distances between atoms and the extent of overlap within the electron cloud, consequently affecting the transmission pathways and efficiency of electrons [36]. Furthermore, lattice distortion introduces defects, which often serve as active sites that enhance the adsorption capacity of reactants and facilitate the formation and transformation of reaction intermediates, thereby improving catalytic activity.

**Figure 3:**
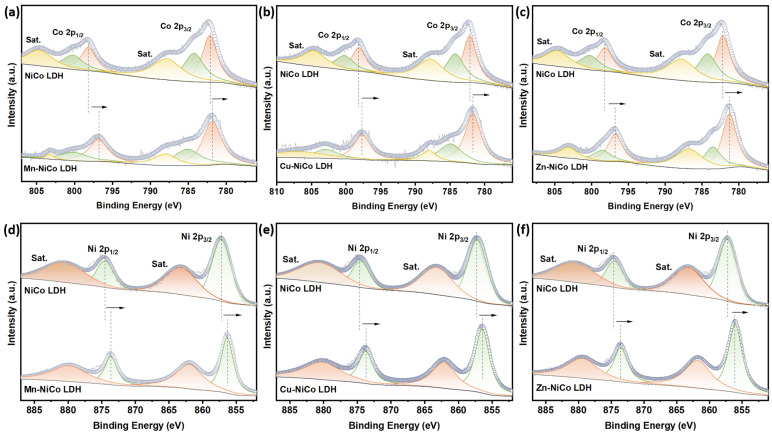
Co 2p XPS of (**a**) Mn-NiCo LDH, (**b**) Cu-NiCo LDH, and (**c**) Zn-NiCo LDH. Ni 2p XPS of (**d**) Mn-NiCo LDH, (**e**) Cu-NiCo LDH, and (**f**) Zn-NiCo LDH.

High-resolution X-ray photoelectron spectroscopy (XPS) emerged as the analytical tool that precisely determined the chemical states of elements. To guarantee the precision and dependability of the XPS data, the peak position of every element was calibrated with the aid of the C 1s peak at 284.8 eV. As depicted in Figure 2b, the high-resolution O 1s spectra acquired from all experimental samples exhibit a remarkable degree of complexity, allowing for a detailed analysis through the process of deconvolution. Specifically, these spectra can be effectively partitioned into three distinct peaks, each of which corresponds to different chemical states of oxygen present within the samples. In the case of the NiCo LDH, the peak at 533.30 eV is related to oxygen in the adsorbed state. The peak at 532.20 eV is oxygen vacancies (O_v_). Meanwhile, the peak at 531.45 eV is combined with metal–oxygen bonding (M-O) [37]. These oxygen vacancies may enhance the adsorption of OH^−^ by providing extra adsorption sites [38]. In all the doped samples, the oxygen vacancies showed a slight negative shift compared with the NiCo LDH. This observed shift, within the context of the chemical and physical phenomena under study, implies a rather significant difference in the electronic environment that surrounds the oxygen atoms, which may be related to the interaction between the doping elements and the host NiCo LDH structure. The high-resolution Ni 2p XPS spectrum (Figure 2c) exhibits two characteristic spin–orbit split components corresponding to Ni 2p_3/2_ (857.1 eV) and Ni 2p_1/2_ (874.7 eV) states, demonstrating a peak separation of 17.6 eV, fitting with reference data for Ni^2^^+^ species in NiCo-LDH systems. The deconvolution of the Co 2p XPS spectrum (Figure 2d) reveals dual oxidation states, with resolved components at 784.28 eV (2p_3/2_) and 800.37 eV (2p_1/2_), characteristic of Co^2+^ species, contrasted by corresponding Co^3+^ signals observed at 782.18 eV (2p_3/2_) and 798.14 eV (2p_1/2_). This distinct separation between divalent and trivalent cobalt states aligns with established spectral fingerprints for NiCo-LDH systems. Obviously, compared with the NiCo LDH, both Ni 2p_3/2_ and Co 2p_3/2_ exhibit a negative shift not only in the Fe-NiCo LDH but in the other samples (Figure 2c,d and Figure 3a–f). The spin–orbit splitting magnitudes and principal peak locations served as diagnostic markers for identifying nickel–cobalt oxidation states while simultaneously revealing electronic configuration modifications induced by cationic substitution. The observed spectral shifts demonstrate the presence of intercomponent electronic interactions, which enable the precise modulation of the electron density distribution in the Fe-NiCo LDH and analogous heteroatom-modified systems. Such controlled electronic restructuring favors the generation of catalytically active centers while optimizing interfacial electron transport pathways between the electrode surface and oxygen evolution reaction (OER) intermediates [39]. After doping, the electron binding energies of Ni and Co changed significantly, intuitively demonstrating a modification in the electronic structure. Specifically, the d-orbital hybridization between Fe and neighboring Ni/Co atoms enhances the localized electron density at active sites [40]. This enhancement is advantageous for the adsorption of OH^−^ and the subsequent electron transfer process, thereby promoting the OER reaction.

**Figure 4:**
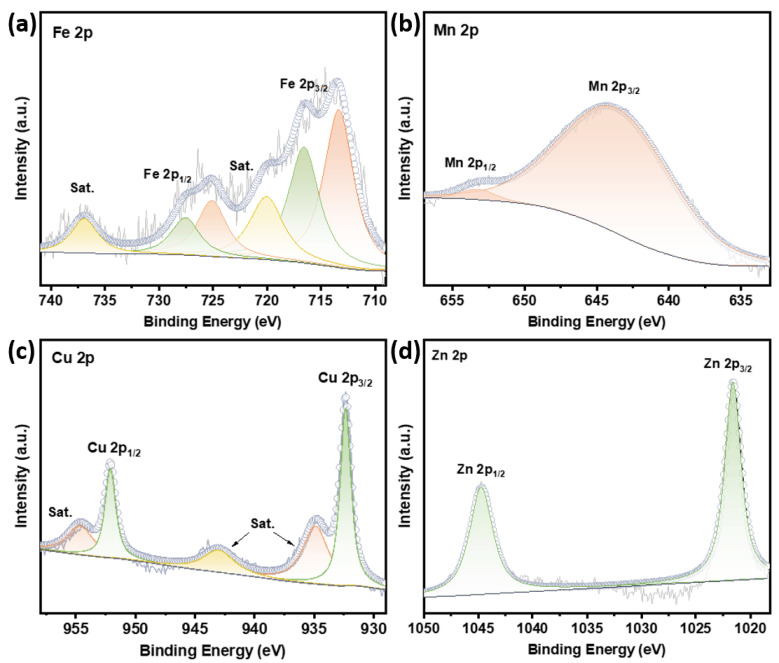
(**a**) Fe 2p XPS of Fe-NiCo LDH. (**b**) Mn 2p XPS of Mn-NiCo LDH. (**c**) Cu 2p XPS of Cu-NiCo LDH. (**d**) Zn 2p XPS of Zn-NiCo LDH.

As shown in the Fe 2p spectra of the Fe-NiCo LDH (Figure 4a), the two peaks corresponding to Fe^3+^ are at 716.6 and 727.6 eV, while the peaks for Fe^2+^ are at 713.2 and 725.1 eV. The associated satellite peaks are located at around 720.1 and 736.9 eV. The spectral deconvolution of the Mn-NiCo LDH (Figure 4b) identifies Mn^2+^ signatures through 2p_3/2_ (644.3 eV) and 2p_1/2_ (653.3 eV) transitions in the Mn 2p region. In Cu 2p spectra of the Cu-NiCo LDH (Figure 4c), the peaks at around 932.3 and 952.1 eV are Cu 2p_3/2_ and Cu 2p _1/2_ of Cu^2+^, respectively. The associated satellite peaks are located at around 934.9, 943.1, and 954.7 eV of strong Cu^2+^. The Zn 2p XPS spectra of the Zn-NiCo LDH are shown in Figure 4d; the two peaks at 1021.5 and 1044.6 eV are Zn 2p_3/2_ and Zn 2p _1/2_ of Zn^2+^, respectively. In the doped catalyst, the binding energy of Fe, Mn, Cu, and Zn (Figure 4a–d) increased compared to the general oxides. This increase may be attributed to the heightened electron cloud density surrounding the Ni and Co elements. To maintain charge balance, the electron cloud density around Fe, Mn, Cu, and Zn consequently decreased. Notably, Fe exhibits two distinct oxidation states: divalent and trivalent. The rise in binding energy contributes significantly to the stability of Fe^2+^ ions. This enhanced stability could facilitate the processes of adsorption and desorption for intermediates involved in the reactions. Furthermore, the stability of Fe^2+^ has implications for the overall reaction mechanism of the OER. A more stable Fe^2+^ can alter the pathway and kinetics of the OER, potentially leading to improved performance in catalytic applications. A more stable Fe^2+^ can act as a more efficient electron transfer mediator [41]. During the OER, the transfer of electrons between different oxidation states of Fe can improve the formation and transformation of reaction intermediates. Fe^2+^ can be oxidized to Fe^3+^ during the reaction, and then Fe^3+^ can accept electrons from the catalyst surface and be reduced back to Fe^2+^, completing the electron transfer cycle. The enhanced stability of Fe^2+^ can ensure the smooth progress of this cycle, improving the overall catalytic performance [42].

**Figure 5:**
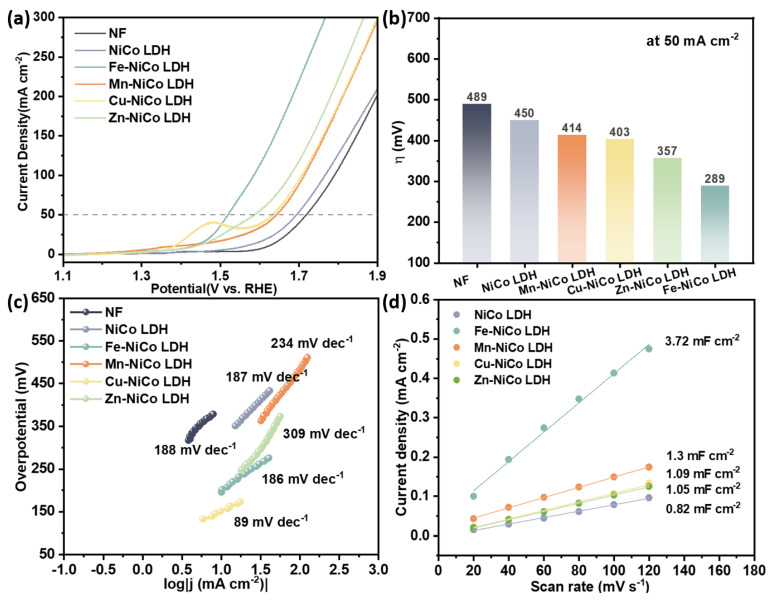
(**a**) LSV curves, (**b**) *η* at a current density of 50 mA cm^−2^, (**c**) Tafel plots, and (**d**) linear plots of current density (Δj/2) versus scan rate for NiCo LDH, Fe-NiCo LDH, Mn-NiCo LDH, Cu-NiCo LDH, and Zn-NiCo LDH.

The catalytic activity of the materials investigated for the OER was meticulously within an alkaline environment, using a 1.0 M potassium hydroxide (KOH) solution. The LSV curves for the catalysts are illustrated in Figure 5a. The Fe-NiCo LDH catalysts show remarkable electrocatalytic activity for the OER, with an overpotential of merely 289 mV at 50 mA cm^−2^, as depicted in Figure 5b. In contrast, the undoped NiCo LDH shows higher overpotential (450 mV) at the same condition. The significant difference in overpotential clearly demonstrates a remarkable enhancement in the catalytic performance of the Fe-NiCo LDH. At a current density of 50 mA cm^−2^, other doped samples, such as the Mn-NiCo LDH (414 mV), Cu-NiCo LDH (403 mV), and Zn-NiCo LDH (357 mV), also display lower overpotentials when compared to the undoped NiCo LDH. This clearly shows that doping positively affects the modification to the NiCo LDH for the OER.

The incorporation of Fe atoms into the NiCo LDH modifies its electronic structure, which in turn improves the capacity of active sites to capture hydroxide ions (OH^−^). This improvement in adsorption capacity facilitates a more rapid reaction rate in the OER. A detailed analysis using XPS (Figure 2c,d) demonstrated that the electron binding energies for both Ni and Co registered notable changes following the doping process with Fe. This observation serves as compelling evidence of the alteration in the material’s electronic structure. More specifically, the interaction between the d-orbital electrons of Fe and those of Ni and Co results in an increase in the electron cloud density surrounding the active sites, thereby promoting enhanced catalytic activity [43]. This improvement aids in the adsorption of OH^−^ ions and facilitates the subsequent electron transfer process, thereby significantly promoting the OER. The catalytic activity of the NiCo LDH is greatly increased through metal doping. Considering the fundamental catalytic characteristics of the NiCo LDH, the incorporation of dopants brings about diverse effects that further impact its overall performance. Coupled with the SEM analysis (Figure 1c,d), it is clear that the nanoflower structure of the Fe-NiCo LDH demonstrates the most significant performance optimization. Although the thorn-like snowflake structure of the Zn-doped NiCo LDH is different from the layered nanoflower structure of the Fe-NiCo LDH, it significantly reduces the overpotential. The surfaces of the stick exhibit roughness with non-uniformed spots (Figure 1i,j). The rough and non-uniform surface of the thorns in Zn-NiCo LDH benefits not only the surface area for OH^−^ adsorption but the micro-environments with different electrochemical properties. These micro-environments can improve the charge transfer process and the adsorption–desorption of reaction intermediates. From an electronic structure perspective, Zn doping may adjust the energy levels of the valence and conduction bands in the NiCo LDH. This adjustment can optimize the interaction between the catalyst and the reactants, promoting the OE. Zn doping might lower the energy barrier for the electron transfer from the catalyst to the adsorbed OH^−^ species, thus accelerating the oxidation process [44]. While this structure is distinctly different from the original layered configuration, it nonetheless contributes positively to performance enhancement. Although the Mn-NiCo LDH and Cu-NiCo LDH have higher overpotentials than the Fe-NiCo LDH and Zn-NiCo LDH, they still show significant improvement compared to the undoped NiCo LDH. This indicates that even though their morphologies and electronic structure modifications are not as effective as those of the Fe-NiCo LDH and Zn-NiCo LDH, the doping still positively impacts the OER activity. For the Mn-NiCo LDH, Mn doping can introduce new electronic states in the NiCo LDH. These new states can act as additional active sites or modify the existing active site properties. The variable oxidation states of Mn can participate in the redox reactions during the OER, improving the transfer of electrons [45]. However, the less-ordered morphology of the Mn-NiCo LDH might limit the accessibility of these active sites, resulting in a relatively higher overpotential when compared with the Fe- or Zn-doped NiCo LDHs. The Cu-NiCo LDH also benefits from the doping effect. Cu is known for its good electrical conductivity and ability to participate in redox reactions. When doped into the NiCo LDH, Cu can enhance the efficiency of the charge transformation within the catalyst. The Cu atoms can act as electron transport centers, increasing the movement of electrons from the bulk of the catalyst to the surface where the OER occurs.

The OER kinetics of the catalysts were evaluated by obtaining the Tafel slopes from the LSV curves (Figure 5c). The Tafel slope for the Cu-NiCo LDH was measured at 89 mV dec^−1^, which is lower than that of the other samples, suggesting that the Cu-NiCo LDH has more rapid OER kinetics. A smaller Tafel slope implies that the reaction rate is more sensitive to the overpotential. Compared to the other catalysts, for the Cu-NiCo LDH, a relatively minor increment in the overpotential is capable of bringing about a substantial rise in the current density. Additionally, the LSV curves image (Figure 5a) shows that at 10 mA cm^−2^, the overpotential of the Cu-NiCo LDH is the lowest. This reduction in the overpotential may be attributed to the doping of Cu, which enhances electron transport and minimizes energy loss during electron transmission [46]. Cu doping is likely to induce alterations in the reaction pathway. In the course of the OER process, reactions can progress via diverse intermediates. The existence of Cu might facilitate the generation of particular reactive intermediates. As a result, the activation energy of the reaction is decreased, and the reaction kinetics are enhanced. One possible explanation is that Cu can interact with OH^−^ and other reaction species to form a more reactive complex. This complex can then be more easily converted to the final product, O_2_. Cu may form a Cu-OH bridge that has a lower energy barrier for the subsequent oxidation steps compared to the reaction pathway without Cu. Another point to consider is that doping with Cu can also have an impact on the surface charge distribution of the catalyst. A more favorable distribution can enhance the adsorption of OH^−^ and improve the desorption of reaction products, such as O_2_, which can further improve the reaction kinetics.

In electrochemical catalysis, the double-layer capacitance (C_dl_) is directly proportional to the electrochemically active surface area (ECSA) of the catalyst, which can be estimated by determining C_dl_ (Figure 5d). The Fe-NiCo LDH (3.72 mF cm^−2^) has a larger C_dl_ value compared with the NiCo LDH (0.82 mF cm^−2^), Mn-NiCo LDH (1.3 mF cm^−2^), Cu-NiCo LDH (1.09 mF cm^−2^), and Zn-NiCo LDH (1.05 mF cm^−2^). The larger C_dl_ value of the Fe-NiCo LDH indicates a larger ECSA, meaning there are more exposed active sites on its surface. The unique 3D layered nanoflower structure of the Fe NiCo LDH is likely responsible for this large ECSA. The hierarchical nanoflower structure provides a high surface-to-volume ratio, allowing for a greater number of active sites to be exposed to the electrolyte. These active sites can interact with OH^−^ or other intermedia more effectively. The increased number of active sites leads to more available reaction sites, allowing for complete contact with electrolytes. Consequently, this promotes the reaction and enhances the reactivity of the catalyst. The enhanced performance of the Fe-doped NiCo LDH compared to the other dopants can be attributed to several key mechanisms. First, the incorporation of Fe into the NiCo LDH structure leads to significant changes in the electronic structure of the catalyst. Fe has a higher electron density and a different electronegativity compared to Ni and Co, which results in a redistribution of the electron cloud density around the active sites. This redistribution enhances the adsorption of hydroxide ions (OH^−^) and facilitates the electron transfer process during the OER. Second, the Fe-doped NiCo LDH exhibits a unique 3D layered nanoflower structure, which provides a large surface area for reactant adsorption and efficient charge transfer. The hierarchical arrangement of the nanoflowers allows for better exposure of active sites and improved mass transport, which are crucial for enhancing the catalytic activity. When compared with the undoped NiCo LDH, the Mn-, Cu-, and Zn-NiCo LDHs exhibit larger C_dl_ values. This phenomenon implies that doping is able to enhance the ECSA of the NiCo LDH. Regarding the Mn-NiCo LDH, despite its morphology being less regular than that of the Fe-NiCo LDH, Mn doping can still give rise to new active sites or uncover more active sites on the surface. Mn atoms can introduce defects or modify the surface structure, leading to an increase in the ECSA. The Cu-NiCo LDH and Zn-NiCo LDH also benefit from doping in terms of the ECSA. Cu and Zn atoms can disrupt the original lattice structure of the NiCo LDH, creating more surface active sites. The unique morphology of the Zn-NiCo LDH, although different from that of the Fe-NiCo LDH, also contributes to the increase in the ECSA. The 3D thorn-like snowflake structure of the Zn-NiCo LDH provides a large surface area, which is reflected in its relatively large C_dl_ value.

## 4. Conclusions

In this study, a series of transition-metal-doped NiCo LDH catalysts were successfully synthesized via the one-step hydrothermal method. The doping of Fe, Mn, Cu, and Zn significantly enhanced the oxygen evolution reaction (OER) performance. At a current density of 50 mA/cm^2^, the undoped NiCo LDH had an overpotential of 450 mV, while the Fe-doped NiCo LDH showed a remarkable improvement, achieving an overpotential of 289 mV. The Mn-, Cu-, and Zn-NiCo LDHs also exhibited lower overpotentials of 414 mV, 403 mV, and 357 mV, respectively, at this current density. The doping modified the morphology, crystal structure, and electronic structure of the NiCo LDH. There was a unique 3D layered nanoflower in the Fe-NiCo LDH and a 3D thorn-like snowflake in the Zn-NiCo LDH, both of which increased the surface area for reactant adsorption. XRD analysis revealed that Fe and Mn doping led to lattice distortions, changing the lattice parameters. XPS revealed that doping altered the chemical states of elements and the electronic environment around them. In terms of electrochemical properties, the electrochemically active surface area, estimated by the double-layer capacitance (C_dl_), increased in doped samples. The Fe-doped NiCo LDH had a C_dl_ value of 3.72 mF/cm^2^, where the undoped NiCo LDH had a value of 0.82 mF/cm^2^. The Cu-NiCo LDH exhibited the fastest OER kinetics with a Tafel slope of 89 mV dec^−1^.

This study suggests several potential avenues for future research to further enhance OER efficiency. Future work could explore the doping of NiCo LDHs with other transition metals, such as Mo, W, or V, to identify new catalysts with even higher OER performance. The development of composite materials, such as NiCo LDHs combined with conductive polymers or carbon-based materials, could improve the electronic conductivity and stability of the catalysts. The use of advanced characterization techniques, such as in situ X-ray photoelectron spectroscopy (XPS) and X-ray absorption spectroscopy (XAS), could provide deeper insights into the electronic and geometric structures of the catalysts during the OER process. In conclusion, this study demonstrates the potential of transition-metal-doped NiCo LDH catalysts for efficient OER and provides a foundation for future research to further enhance the performance and sustainability of these catalysts.

## Data Availability

The original contributions presented in the study are included in the article, further inquiries can be directed to the corresponding author.

## Abbreviations and Full Forms

LDH: layered double hydroxide; OER: oxygen evolution reaction; ECSA: electrochemically active surface area; C_dl_: double-layer capacitance; SEM: Scanning Electron Microscope; XRD: X-ray diffraction; XPS: X-ray photoelectron spectroscopy; LSV: linear sweep voltammetry; Tafel: Tafel slope; RHE: reversible hydrogen electrode; NF: nickel foam; M-NiCo LDH: transition-metal-doped nickel–cobalt layered double hydroxide (where M represents Fe, Mn, Cu, or Zn).
